# P07-10 Nice acti'santé: Let’s move for health in Nice

**DOI:** 10.1093/eurpub/ckac095.110

**Published:** 2022-08-29

**Authors:** Charlène Falzon, Aurélien Lazzaro, Emmanuelle Soummer, Barbara Prot, Richard Chemla

**Affiliations:** Ville de Nice, France

**Keywords:** chronic disease, health, evaluation, orientation, follow-up

## Abstract

**Issue:**

In view of the multiple benefits, the integration of physical activity into the health care pathway of people with chronic diseases is a major issue. In Nice, nearly a third of the population is over 60 years old and more than 60,000 people are in long-term illness. In view of this, the City of Nice has decided to set up a physical activity prescription device named Nice Acti'Santé to support people in Nice with a chronic illness who want to make physical activity part of their life.

**Description of the problem:**

The main objective of the device is to encourage people with a chronic disease to engage in a physical activity appropriate to their health. A medico-active process has been implemented to meet this objective: (a) the doctor guides his patient toward the Nice Acti'Santé platform, (b) the platform team makes a free checkup of the patient and guides him toward a partner sports club, (c) the doctor validates the patient's orientation, prescribes the physical activity and signs the certificate, (d) the partner sports club takes charge of the patient and the plateform team sets up a follow-up, (e) after 4 months, the platform team makes a new checkup of the patient and guides him toward an autonomous practice and, (f) the doctor validates the new patient's orientation and signs the certificate.

**Results:**

Preliminary results show that 1 out of 2 patients is registered in a partner sports club after being taken charge on the platform. Most patients are aware of the platform through media or communication supports, and few are oriented by their doctor.

**Lessons:**

The platform (a) acts in complementarity with the doctor who can count on a quality device with qualified staff to take charge of his patient, (b) guides the patient toward a physical activity adapted to health and taking account his capacities and needs and, (c) helps partner club sports to recruit participants.

**Main messages:**

Nice Acti'Santé is an effective link between health and sport professionals/Nice Acti'Santé is an innovative and local device to serve the most vulnerable people in Nice.

